# Bacteriological profile, antimicrobial susceptibility patterns of the isolates among street vended foods and hygienic practice of vendors in Gondar town, Northwest Ethiopia: a cross sectional study

**DOI:** 10.1186/s12866-019-1509-4

**Published:** 2019-06-07

**Authors:** Azanaw Amare, Tifito Worku, Birukitait Ashagirie, Marie Adugna, Alem Getaneh, Mulat Dagnew

**Affiliations:** 1 Department of Medical Microbiology, University of Gondar, College of Medicine and Health Sciences School of Biomedical and Laboratory Sciences, Gondar, Ethiopia; 2 University of Gondar, College of Medicine and Health Sciences School of Biomedical and Laboratory Sciences, Comprehensive Specialized Hospital Laboratory, Gondar, Ethiopia

**Keywords:** Street vended foods, Bacterial profile, Antibiotic susceptibility patterns, Gondar town

## Abstract

**Background:**

There are numerous advantages offered by street vended foods, but evidence exists that foods exposed for sale on the road side may be contaminated by pathogenic microorganisms. However, information on the bacteriological profile, bacterial load and antimicrobial susceptibility patterns of bacterial isolates from street food in Gondar town are lacking. The aim of this study was to assess bacterial profile, bacterial load, and antimicrobial susceptibility patterns of bacterial isolates among street vended foods and also the hygienic practice of vendors in Gondar town, Northwest Ethiopia.

**Methods:**

Socio-demographic characteristics and the hygienic practices of 24 vendors were collected using structured questionnaire. A total of 72 food samples from four different food items were analyzed and counted by standard aerobic plate count method. Ten grams of each food sample was transferred in to 90 ml of buffered peptone water and homogenized. The homogenates were serially dilute and a volume of 0.1 ml dilution was spread on solid media and incubated at 35-37 °C for 24 h. Antibiotic susceptibility testing was done for isolated species using Muller Hinton agar and data was entered and analyzed by using SPSS version 20.0.

**Results:**

Seventy two food samples of street vended food were analysed for bacterial pathogens. 44/72 tested positive, a total of 63 isolates were identified as 19 samples contained two pathogens. The total mean aerobic bacterial count was 6.64 × 10^4^ CFU/g which is varied from 1 × 10^4^–1.86 × 10^5^ CFU/g. *S. aureus is* the most frequent isolate 34 *(*53.96%) followed by *E.coli* 15(23.8%), *Enterobacter species* 10(15.87%) and *Citrobacter species* 4(6.3%). Gentamycin, chloramphenicol, tetracycline, ciprofloxacin, and trimethoprim-sulfamethoxazole were found to be the most effective antimicrobials against all isolates but the enterobactereaceae were resistant to ampicillin and Ceftaziidime and *S.aureus* were resistant to penicillin.

**Conclusion:**

The results of this study showed that, the majority of street-vended food items in Gondar were contaminated with one or more different pathogenic bacteria. The presence of these bacteria in foods could lead to potential health problems for consumers. Therefore, health education as well as training in food safety and hygienic handling is required for food handlers to minimize contamination and the likelihood of people falling ill.

## Background

Street vended food is defined according to Food and Agriculture Organization (FAO) as ‘ready to eat foods and beverages prepared and sold by vendors especially in streets and similar public places for immediate consumption without further processing or preparation’ [1]. Though the street vended food plays an important role both economically and socially in meeting food demands in urban people [2], microbiologically contaminated street food is considered a global problem, which is liable to be a significant contributor to the transmission of food borne diseases [3, 4]. Food borne diseases causes high morbidity mainly in developing countries [5, 6]; due to poor hygienic condition during food preparation and the lack of awareness about food safety [3, 7]. According to a 2017 WHO, report the global burden of food borne diseases states that each year as many as 600 million, or almost 1 in 10 people in the world, fall ill after consuming contaminated food. Of these, 420,000 people die, including 125,000 children under the age of 5 years [8]. However, in most of developing countries, data regarding food borne illnesses remain scarce. With cross border movement of people and food ingredients rapid identification of a problem in one country could prevent further illness in another if the incident is communicated quickly through an organized system [9, 10].

Unhygienic practices substantially contributing to the entry of bacterial pathogens to food include contamination from surfaces of raw materials and equipment, improper storage or refrigeration, and unsanitary conditions of surfaces in the working environment [11]. Street foods pose a risk to public health because they are openly displayed and are exposed to dust, insects, and the hands of the food handlers and customers [12].

In addition potable tap water is often not available at the vending site except occasionally one or more buckets of water for washing hands and utensils sometimes without soap. Moreover, sanitary facilities are rarely available for workers. Wastes are disposed of nearby, providing nutrients for flies and rodents and this may harbour food borne pathogens [13]. As a result, ready to eat prepared street foods are commonly exposed to a variety of potential public health risks. Contamination with pathogenic microorganisms like *E. coli, Salmonella, Shigella, Campylobacter* and *S. aureus* [14, 15] may occur during preparation, post-cooking and at various handling stages [16, 17]. Various studies report that food borne diseases associated with the consumption of contaminated food from different vendors in India [18], Mexico [19], and Ethiopia [20]. Food borne pathogens for example, *Salmonella* and *Shigella* were identified in similar studies of street vended foods in different places of Ethiopia [17, 21].

In addition, food borne illness can also be caused by bacterial toxins from *Staphylococcus aureus, Clostridium botulinum* and *Bacillus cereus* primarily transmitted by food contaminated due to unhygienic handling [22, 23].

Knowing the bacteriological quality of street vended foods is an important factor in recognizing the safety problems relate to street foods. Evidence of laboratory based diagnosis on the assessment of bacteriological profile and their antimicrobial susceptibility pattern is important for designing and implementing effective treatment, and control and prevention strategies to effectively tackle the problem. In Gondar town manual workers and other low income individuals use street food as it is both available and affordable. However, the quality of these foods and their safety for human health is not well known. Some limited research has been conducted on their quality in relation with the bacterial load and associated risk factors but antimicrobial susceptibility was not performed. Therefore, this study was conducted to provide additional information to inform policies to protect public health. Street food is widespread throughout Ethiopia and the findings from this study are also applicable to other areas.

## Methods

### Study area and setting

The study was conducted in six areas (College area, Arada, Piazza, Hospital, Biliko and Azezo) of Gondar town, Northwest Ethiopia where there are resident populations are high and street vendors are common. Gondar is located at 737 km from the country’s capital Addis Ababa in North Gondar zone of the Amhara Regional State.

### Study design and period

Across-sectional study was conducted from January 2017 to May 2017.

### Sample size and sampling technique

The study included twenty four street food vendors for data collection. Four frequently vended and highly consumer foods were selected for sampling: Sanbusa, Donat, Bombolino and bread from six vending sites. A total of 72 food samples (three food samples from each food type from each selected site) were collected aseptically and analyzed (Table 1).

**Table 1:** The ingredients and description of street foods analysed from Gondar town

Food items	Ingredients	Description
‘Donat’	Wheat dough, Salt, cooking oil, baking powder, Sugar, Sweeteners and flavorings like jam, custard or cream.	It is a deep fried piece of wheat dough commonly of a circular or flattened sphere shape covered with jam, custard or cream.
‘Bombolino’	Wheat dough, cooking oil, baking powder, salt, Sugar (optional)	It is a fried piece of wheat dough commonly of a ring or circular shape without jam, custard or cream.
‘Sanbusa’	Wheat dough, salt, bread spices, onion, cooking oil, rice, lentil.	It is a deep fried piece of wheat dough of a triangle or distinctive shape stuffed with chopped onion, lentils or rice.

### Data collection

Data related to socio-demographic characteristics and personal hygiene practices of food handlers were collected by face to face interview using pre tested structured questionnaire and checklist. All the questionnaires were checked for accuracy and completeness. The questionnaire and checklist were prepared in English version and translated to Amharic version which is the local language of the study participants.

### Sample collection and processing

Approximately 100 g of food sample was collected aseptically using sterile aluminum foil from each item from the original closed container and transported (using box that contain ice pack) to the medical microbiology laboratory, University of Gondar. The food samples were cut into smaller pieces using surgical blade and picked by sterile forceps and measured 10 g using digital balance. The measured sample was homogenized in a measured volume (90 ml) of 0.1% buffered peptone water (BPW). The homogenate was further serially diluted by mixing one part of homogenate with nine parts of diluents to have serial dilution up to10 ^− 6^ using test tubes and then transferred into the culture media which is prepared according to the manufacturer’s instruction [24, 25]. After mixing each tube, 0.1 ml suspension was transferred and spread using wire loop on to a sterile plate count agar (PCA) in duplicate for total aerobic plate count and to MacConkey (Mac) agar, Mannitol salt agar (MSA) and *Salmonella Shigella* agar (SSA) for *Enterobacteriaceae*, S*taphylococcal* and *Salmonella-Shigella* count respectively [25]. The plates then incubated at 35-37^o^ C for 18–24 h for bacterial growth.

### Bacterial isolation, identification, and enumeration

The results of each plate having colonies between 30 and 300 were counted using colony counter SC6. Average counts obtained expressed as Colony Forming Units per gram of food (CFU/g) by multiplying the number of bacteria by the dilution factor [26]. Gram stain was done from bacterial colonies. Selected isolates from Mac and SSA then sub-cultured into nutrient agar and were incubated to make the sample refresh for different bio chemical tests. Colonies were inoculated in to the following biochemical tests for identification of Gram negative bacteria: Triple sugar iron (TSI) agar, Lysine Decarboxylase (LDC), Simmons Citrate, Urea and Sulfide Indole Motility (SIM). The inoculated biochemical tests were incubated for 18–24 h at 35-37 °C and were checked for any color change [27]. Subculture of the isolate that grew on MSA and nutrient agar: Gram stain and appropriate biochemical tests (Catalase and coagulase test) were undertaken [28–30].

### Antimicrobial susceptibility testing

Antimicrobial susceptibility testing (AST) were checked for isolates by using Modified Kirby Bauer Disk Diffusion method using Mueller-Hinton agar (MHA). It was performed according to the Clinical and Laboratory Standards Institute (CLSI) guidelines [31]. Three to five colonies of bacteria were taken and transferred to a tube containing 5 ml normal saline and mixed gently until a homogenous suspension formed. Turbidity of the bacterial suspension was compared with 0.5 McFarland turbidity standards. The bacterial suspension was then swabbed over the entire surface of MHA using a sterile cotton swab. Antibiotic discs were applied within fifteen minutes using sterile forceps on the surface of medium and incubated at 35-37 °C for 18–24 h. The zone of inhibition of growth around each disk was then measured in millimetres and zone diameters interpreted in accordance with standards as sensitive, intermediate and resistant. The antibiotic discs tested for isolate of bacterial species include: clindamycin (25 μg), tetracycline (30 μg), norfloxacin (10 μg), ampicillin (10 μg), ciprofloxacin (5 μg), cotrimoxazole (25 μg), ceftriaxone (30 μg), chloramphenicol (25μg), gentamicin (10 μg), ceftazidime (25 μg), ceftriaxone (25 μg) and penicillin (10 μg).

### Data quality control

Aseptic technique was used throughout all sampling and handling procedures by using sterile materials, flaming and refrigeration. To avoid unpredictable changes, samples were analyzed without delay and identical samples were analyzed three times in order to confirm the contamination levels. For significant studies of microorganism, pure culture was used. Receptacles containing all essential nutrients were free prior sterilized; solution and equipment containing water were autoclaved at 121^O^c for 15 to 20 min. The sterility of the media were checked by incubating 5% of the batch at 37 °C for 18–24 h, and icebox was used during sample collection and transportation and the performance of the media were checked using *S.aureus* (ATCC, 25923) for MSA and *E. coli* (ATCC, 25922) for MAC.

### Operational definitions

#### Street food

A ready-to-eat food sold by vendor in a street or other public place, such as at a market.

#### Ready to eat foods

Foods that are eaten directly without further preparation.

#### Street food vendors

Persons who offer food for sale on the street to the public without having a permanently built structure but with a temporary structure or mobile facility.

#### Data analysis and interpretation

Data were cleaned manually, entered and analyzed by using SPSS version 20.0. Descriptive statistics was used for analysis and the results were interpreted. Number of colonies equal to10 per gram of *staphylococcus species* were considered potentially hazardous as consumption foods with this level of contamination may result in food borne illness. Ready-to-eat foods should be free from *Salmonella* and *shigella species* as consumption of food containing this pathogen may result in food borne illness [32].

## Results

### Socio demographic characteristics of the study participant

Of the 24 street food vendors recruited in this study, majority (75%) were females. Ten (41.7%) of the vendors were below the age of 25 years, 11 (45.8%) were 26–35 years and 3 (12.5%) were above age of 36 years. Thirteen (54.2%) were primary school completed, 9 (37.5%) secondary school and 2 (8.3%) were college and above.

### Hygienic practice of the study participant

In this study, half of the vendors 12(50%) had no frequent hand washing habit with soap and water during the preparation, collecting and displaying of food. Only 5 (20.8%) of the vendors covered their hair while preparing food and 11 (45.8%) had a frequent nail cutting. In relation to food preparation rooms, 10(41.7%) of the study participants had a separate room for food preparation but the rest were prepared and vended foods in the open space. Eighteen (75%) vendors used cleaned pipe water for food preparation and material cleaning but 6 (25%) were no a clean piped water. With regard to food displaying place we observed that 17(70.8%) displaying sites were clean while the rest were not clean and were exposed to direct sunlight and dust as well as the service was given carelessly with bare hands.

Regarding previous history of food borne illness, even thought the majority 19(79.2%) of the study participants had no training about food safety, few of them 10 (41.7%) reported a history of food borne illness (Table 2).

**Table 2:** Hygienic condition of street food vendors in Gondar town, from January–May 2017

Hygienic condition	Response	Frequency	Percent (%)
Hand washing practice	Yes	12	50.0
	No	12	50.0
Use of hair cover	Yes	5	20.8
	No	19	79.2
Practice of nail cutting	Yes	11	45.8
	No	13	54.2
Separate closing during cooking	Yes	12	50
	No	12	50
Separate kitchen availability	Yes	10	41.7
	No	14	58.3
Reservation for food utensils safety	Yes	19	79
	No	5	21
Use of clean water	Yes	18	75
	No	6	25
Selling site cleanness	Yes	17	70.8
	No	7	29.2
No contact of hand with food during vending	Yes	13	54.2
	No	11	45.8
Safety of food from dust, sun and winds	Yes	10	41.7
	No	14	58.3
Safety of food transportation	Yes	16	66.7
	No	8	33.3
Training on food safety	Yes	5	20.8
	No	19	79.2
History of food borne diseases	Yes	10	41.7
	No	14	58.3
Information on food born diseases	Yes	10	41.7
	No	14	58.3
Availability of toilet	Yes	22	91.7
	No	2	8.3
Presence of safe water pipe	Yes	23	95.2
	No	1	4.8
Presence of waste pit	Yes	14	58.3
	No	10	41.7

### Bacterial profile and isolated species in selected foods

A total of 72 street vended food samples were analyzed for the presence of bacterial pathogens. The study revealed 44/72 (61.1%) of the food samples had bacterial contamination. The total mean aerobic bacterial count (ABC) was 6.64 × 10^4^ CFU/g which is varied from 1 × 10^4^–1.86 × 10^5^ CFU/g. The total mean *Enterobacteriaceae* count (MEC) was 5.55X10^4^ CFU/g in which the value ranged from 1.65 × 10^2^–1.79 × 10^5^ CFU/g and the total mean *Staphylococcal* count (MSC) was 5.96X10^4^ CFU/g which varied from 1.25 × 10^4^-2 × 10^5^ CFU/g (Table 3).

**Table 3:** Mean bacterial count from food Samples in Gondar Town from January 2017 to May 2017

Food item	Aerobic Bacterial Count			*Enterobacteriacae* count			*Staphylococcal* Count		
	Min	Max	Average	Min	Max	Average	Min	Max	Average
Sanbusa	1 × 10^4^	1.86 × 10^5^	6.45 × 10^4^	1.65 × 10^2^	1.25 × 10^5^	2.29 × 10^4^	1.25 × 10^4^	2 × 10^5^	4.43 × 10^4^
Donat	1.2 × 10^4^	1.78 × 10^5^	5.55 × 10^4^	1.7 × 10^3^	1.97 × 10^4^	1.46 × 10^4^	1.36 × 10^4^	1.93 × 10^5^	7.62 × 10^4^
Bombolino	1.2 × 10^4^	1.82 × 10^5^	6.42 × 10^4^	1.2 × 10^4^	1.49 × 10^5^	3.64x10^4^	1.4 × 10^4^	1.65 × 10^5^	5.25 × 10^4^
Bread	1.8 × 10^4^	1.37 × 10^5^	5.78 × 10^4^	1.79 × 10^5^	1.79 × 10^5^	1.79 × 10^5^	1.5 × 10^4^	2 × 10^5^	6.2 × 10^4^
	Total MABC = 6.64X10^4^			Total MEC = 5.55X10^4^			Total MSC = 5.96X10^4^		

*MABC* Mean Aerobic Bacterial Count, *MEC* Mean *Enterobacteriacae* count, *MSC* Mean *Staphylococcal* Count

From 44 contaminated food samples 63 isolates were identified. *S. aureus* was the most frequent isolate 34 *(*53.96%) followed by *E.coli* 15(23.8%), *Enterobacter species* 10(15.87%) and *Citrobacter species* 4(6.3%).The study revealed that 19 of the samples contained two organisms. The highest numbers of bacterial isolates were isolated from sanbusa (25/63) and donat (22/63) while the minimum value was seen in bombolino (10/63) and bread (6/63). In this study, Salmonella and Shigella were not isolated (Table 4).

**Table 4:** Distribution of bacterial isolates from food Samples in different areas in Gondar Town, January–May 2017

		Isolates				Total no. of Isolates	No organism (NO)	Total no of isolates + NO
		*S.aureus*	*E.coli*	*Enterobacter species*	*Citrobacter species*			
Food items	Sanbusa	13	7	4	1	25	2	27
	Donat	13	4	3	2	22	4	26
	Bombolino	4	3	3	0	10	9	19
	Bread	4	1	0	1	6	13	19
Total n(%)		34(37.4)	15(16.5)	10(11)	4(4.4)	63(69.2)	28(30.8)	91(100)
Sample areas	Bilko	4	3	2	0	9	6	15
	Piazza	7	3	1	1	12	4	16
	Arada	8	3	1	1	13	4	17
	Hospital	4	1	2	1	5	5	13
	College	6	4	3	1	14	3	17
	Azezo	5	1	1	0	7	6	13
Total n(%)		34(37.4)	15(16.5)	10(11)	4(4.4)	63(69.2)	28(30.8)	91(100)

### Antibiotic susceptibility pattern

In our study, we have tested isolated bacterial species for their sensitivity pattern against the commonly prescribed antibiotics according to the CLSI guideline. Antibiotic susceptibility of *S.aureus* was tested against 8 commonly prescribed and available antibiotics (tetracycline, ciprofloxacin, trimethoprim-sulfamethoxazole, clindamycin, chloroampinicol gentamycin, and penicillin) using agar disc diffusion method. The results shown that the susceptibility of *S.aureus* isolates was higher to clindamycin (88.23%) followed by tetracycline, gentamycin, ciprofloxacin chloroamphenicol, and trimethoprim-sulfa methoxazole with 85.3, 85.3, 79.4, 79.4, and 61.7% sensitivity respectively. *S. aureus* isolate showed higher resistance to penicillin (73.53%).

In the case of *E.coli* isolates the results shown that the susceptibility was higher for gentamycin (93.33%) followed by ceftriaxone and chloroampinicol each with 86.67% sensitivity. The highest percentage of resistance was recorded against ampicillin (86.67%) and ceftazidime (60%). Enterobacter species had higher sensitivity for gentamycin (90%), ceftriaxone, ciprofloxacin, and tetracycline each with 80% sensitivity but ampicillin (70%) and ceftazidime (70%) showed higher resistance. In the case of citrobacter species 100% was observed on ceftriaxone, tetracyclin and gentamycin but ampicillin (75%) showed higher resistance. In general ciprofloxacin, tetracycline and gentamicin were found to be the most effective antimicrobials against all isolates*.* Clindamycin was also more effective against *S.aureus* isolate.

Ampicillin and ceftazidime against enterobactereacea and penicillin against *S. aureus* isolate were not effective as shown in (Table 5).

**Table 5:** Antibiotic susceptibility patterns of bacterial isolates from food sample in Gondar town, January–May 2017

Antibiotic susceptibility patterns of isolates		Isolates			
		*S. aureus* n (%)	*E.coli* n (%)	*Enterobacter* n (%)	*Citrobacter* n (%)
Ampicillin	S	–	2(13.33)	3(30)	1(25)
	R	–	13(86.67)	7(70)	3(75)
Chloramphenicol	S	27(79.4)	13(86.67)	5(50)	2(50)
	R	7(20.6)	2(13.33)	5(50)	2(50)
Ciprofloxacin	S	27(79.4)	11(73.33)	8(80)	3(75)
	R	7(20.6)	4(26.67)	2(20)	1(25)
Ceftazidime	S	–	6(40)	3(30)	2(50)
	R	–	9(60)	7(70)	2(50)
Tetracycline	S	29(85.3)	12(80)	8(80)	4(100)
	R	5(14.7)	3(20)	2(20)	0
Ceftriaxone	S	–	13(86.67)	8(80)	4(100)
	R	–	2(13.33)	2(20)	0
Penicillin	S	9(26.5)	–	–	–
	R	25(73.5)	–	–	–
Norfloxacin	S	–	7(46.67)	6(60)	2(50)
	R	–	8(53.33)	4(40)	2(50)
Gentamycin	S	29(85.3)	14(93.33)	9(90)	4(100)
	R	5(14.7)	1(6.67)	1(10)	0
Clindamycin	S	30(88.2)	–	–	–
	R	4(11.76)	–	–	–
Trimethoprim-sulfamethoxazole	S	21(61.8)	9(60)	6(60)	3(75)
	R	13(38.2)	6(40)	4(40)	1(25)

*S* Sensitive, *R* Resistance

## Discussion

Street food vending has become an important public health issue owing to the potential to cause food borne diseases. Vendors who lack an adequate understanding of the basic food safety issues mostly display foods sold on the streets [33]. This study showed that majority (75%) of the vendors was young females. The finding is in line with other studies carried out in Ethiopia and elsewhere; Gondar, Addis Ababa, Jigjiga, and Sudan (95, 80, 78.8 and72%) respectively [34–37] and in contrast to a study conducted in Pune, India which is 97.33% of the food vendors were male [38]. In this study all the vendors were literate in contrast to the majority (66.7%) of the vendors in Jigjiga who were illiterate [39] while 42% were illiterates in Khartoum, North Sudan [37]. In this study the majority (58.3%) of the food vendors had no information on food borne diseases in contrast to a previous study conducted in Gondar where 57.5% of the food vendors had knowledge on food borne disease [36].

From the present study it has been found that 45.8%% of the food vendors handled foods with bare hands which is similar to a study conducted in Jigjiga (47.62%) [39]. About 79.2% of the food vendors had no hair covering in contrast to a study from Jigjiga (30.3%) [39]. The majority (75%) of the food vendors used clean water for washing their utensils which is similar to a study conducted in Jigjiga (60.6%) [39]. The majority 19(79.2%) of the study participants had no training in food safety which is similar to a study in the Cameroon where97% of the respondents had not received any training on food safety [40].

In this study the presence of confirmed bacterial pathogens is lower (61.1%) when compared to the previous studies conducted in Gondar (64.3 and 82.8%) [36, 41] and Jigjiga (72%) [39]. This may be due to the street food vendors improving their hygiene.

The MABC (6.64X10^4^ CFU/g) was found to be a little bit different when compared with previous study carried out in Gondar (4.25 × 10^4^ CFU/g) [41]. The bacterial load may be varied from place to place due to many factors which includes environmental condition which is favorable for bacterial proliferation and mainly issue of hygienic measurements to avoid post-contamination of food.

In this study, *S. aureus, E.coli*, *Enterobacter* and *Ctirobacter* species were detected from the four food items. Similar results were reported from the study that was conducted in Harare Zimbabwe, Nairobi Kenya, and Gondar [36, 42, 43]. In our study *S. aureus* were detected in 47.22% and *E.coli* in 20.83% of the food samples. These results are lower than results conducted in Zimbabwe and Gondar, Ethiopia in which 85.5 and 53.7% of the food samples were contaminated by *S. aureus* and 53 and 46.3% of the food samples were contaminated by *E. coli* [36, 42] respectively. This indicates that *S. aureus* is the most predominant bacteria in selected food items followed by *E. coli*. It is known that *E. coli* and *S. aureus* are the most common pathogens found on hands [41, 44]. The hands of the food handlers are the most important vehicle for the transfer of organisms from feces, nose and skin to the food [33]. As a result the food, food related materials, skin, clothes, displaying sites and other materials which are related with poor hygienic condition can be contaminated. Food handler may be cause contamination by poor food handling practices contributing to cross contamination or by being carriers. Besides, some of the foods are left in the pans in which they cooked, until sold or reheated, which can result in longer holding time, hence creating favorable conditions for the growth of *E.coli* and *S. aureus* [33]. The predominance of the two bacteria in street foods is also supported by other studies conducted in Bangladesh, India and Ethiopia [36, 45, 46].

From 34 *S. aureus* isolates the highest detection was observed both in sanbusa and donat [26/72(18%)]. The result indicated that the food samples were highly contaminated from the skin, mouth or nose of food handlers during handling, processing or vending because of poor food handling practices. The finding is in agreement with the previous study carried out in Gondar [34, 41].

The highest detection of *E.coli* was found in sanbusa 7/72 (9.7%). This could be attributed to heat processing failure or post processing contamination, fecals contamination due to poor hygienic practice of food handlers [36, 41].

In this study *Enterobacter* and *Citrobacter* species were also isolated from different food samples. The presence of these organisms in street vended food is confirmed with many other previous studies in Nigeria, Ghana and Kenya [17, 43, 47, 48].

In this study, no *Salmonella* species were encountered. There were other studies in Zimbabwe, Kenya and a previous study in Gondar which are in agreement with this finding [36, 42, 43]. However considering the low sample size together with the fact that enrichment media was not used for isolation, this study cannot prove that street food are free from Salmonella species rather it indicates that the prevalence of Salmonella in street food may be very low. Other various studies in Bahir dar, Congo, Jigjiga and Hawassa show the presence of Salmonella species [7, 17, 39, 49].

From the analyzed food items, bread was the least contaminated food with bacteria. This may be related to the low moisture content [50] and the way of handling and displaying these foods. We observed that they were covered their hands with plastic sheets as well as using long sticks without hand contact, during food storage and serving to customers.

The emergence and re-emergence of antibiotic-resistant food borne bacteria in recent times calls for concerted efforts, especially in developing countries. Children and immune-compromised individuals suffer most from these pathogens [51]. Only Citrobacter species showed 100% sensitivity to gentamicin, ceftriaxone and tetracycline. *S. aureus* showed highest resistance against penicillin (73.53%). *E.coli, Enterobacter* and *Citrobacters species* are resistant to ampicillin (86.67, 70 and 75%) respectively which is comparable with a study conducted in Hawassa (100, 60 and 89% resistance) [17]. Gentamicin (93.33%) was very effective against *E. coli* which is comparable to the findings conducted in Bahir dar [7]. In a study conducted in Hawassa [17] ciprofloxacin was 100% sensitive for *E. coli* and chloramphenicol show highly resistance, but the E coli in this study 73.33% were sensitive to ciprofloxacin and 86.67% to chloramphenicol.

### Limitations of the study

Although this study addresses important things, it is not free from any limitation. Association of risk factors was not done since our main concern was identifying the isolate and doing antimicrobial susceptibility testing. Antimicrobial susceptibility patterns of *S.aureus* using Cefoxitin was not undertaken because of inaccessibility of the appropriate disc and vancomycin susceptibility was not tested as it requires minimum inhibitory concentration but we use a disc diffusion method for all antibiotics. This study also did not address other food borne pathogens due to not using enrichment and other selective media for isolation.

## Conclusion

The results of this study showed that, majority of the street-vended food items in Gondar town were contaminated with one or more different pathogenic bacteria. The presence of these bacteria in foods could lead to potential health problems for consumers at individual and community level as well. Clindamycin, gentamycin, tetracycline, chloramphenicol and ciprofloxacin were found to be the most effective antimicrobials against *S. aureus* isolates, while penicillin was not effective. Gentamycin, ceftriaxone, chloroamphenicol, tetracycline and ciprofloxacin were found to be the most effective antimicrobials against *E.coli,* Enterobacter and *Citrobacter* species, whereas ceftazidime and ampicillin were noteffective against these isolates.

Therefore, there is a requirement for more effective health education and food handling training for street food handlers in Gondar.

## Data Availability

The datasets used and/or analysed during the current study available from the corresponding author on reasonable request.

## Abbreviations

ABC: Aerobic bacterial count
AST: Antimicrobial susceptibility testing
ATCC: American type cell culture
BPW: Buffered peptone water
CFU: Colony Forming Unit
CLSI: Clinical and Laboratory Standards Institute
LDC: Lysine Decarboxylase
MABC: Mean aerobic bacterial count
Mac: MacConkey
MEC: Mean enterobactericeae count
MHA: Miller Hinton Agar
MSA: Manitol Salt Agar
MSC: Mean staphylococal count
PCA: Plate count agar
SIM: Sulfide indole motility
SPSS: Statistical package for social sciences
SSA: Salmonella Shigella Agar
TSI: Tripple sugar iron
WHO: World Health Organization

## References

[1] FAO (1989). Food and Agricultural Organisation. Street foods: A summary of FAO studies and other activities related to street foods.

[2] Cress-Williams L (2001). Food micro-enterprises for food security in an urban slum community in East London: development of an awareness-creating programme.

[3] WHO (1999). Food safety: an essential public health issue for the new millennium. Food Safety Programme, Department of Protection of the Human Environment, Cluster of Sustainable Development and Healthy Environments, World Health Organization, Geneva. 1999.

[4] Al Mamun M, Rahman SMM, Turin TC (2013). Microbiological quality of selected street food items vended by school-based street food vendors in Dhaka, Bangladesh. Int J Food Microbiol.

[5] Ram P, Naheed A, Brooks W, Hossain M, Mintz E, Breiman R (2007). Risk factors for typhoid fever in a slum in Dhaka, Bangladesh. Epidemiol Infect.

[6] Saha SK, Saha S, Shakur S, Hanif M, Habib MA, Datta SK (2009). Community-based cross-sectional seroprevalence study of hepatitis a in Bangladesh. World Journal of: WJG.

[7] Kibret M, Tadesse M (2013). The bacteriological safety and antimicrobial susceptibility of bacteria isolated from street-vended white lupin (Lupinus albus) in Bahir Dar, Ethiopia. Ethiopian J Health Sci.

[8] WHO (2017). WHO report: food safety. 31 October 2017. Available at http://www.who.int/news-room/fact-sheets/detail/food-safety. Accessed 23 July 2018.

[9] Schlundt J (2002). New directions in foodborne disease prevention. Int J Food Microbiol.

[10] WHO (2001). WHO report Global surveillance of food borne disease: Developing a strategy and its interaction with risk analysis. Available at: http://whqlibdoc.who.int/hq/2002/WHO_CDS_CSR_EPH_2002.21.pdf?ua=1. Accessed 24 July 2018.

[11] du Toit L, Venter I. Food practices associated with increased risk of bacterial food-borne disease of female students in self-catering residences at the Cape Peninsula University of Technology. Journal of Consumer Sciences. 2005; 33(1).

[12] Organization WH (1996). Essential safety requirements for street-vended foods.

[13] Tambekar D, Jaiswal V, Dhanorkar D, Gulhane P, Dudhane M (2009). Microbial quality and safety of street vended fruit juices: a case study of Amravati city. Internet J Food Saf.

[14] Guchi B, Ashenafi M. Microbial load, prevalence and antibiograms of Salmonella and Shigella in lettuce and green peppers. Ethiopian journal of health sciences. 2010; 20(1). 10.4314/ejhs.v20i1.69431 PMC3275899 22434959

[15] Tassew H, Abdissa A, Beyene G, Gebre-Selassie S. Microbial flora and food borne pathogens on minced meat and their susceptibility to antimicrobial agents. Ethiopian journal of health sciences. 2010; 20(3). 10.4314/ejhs.v20i3.69442 PMC3275844 22434972

[16] Ghosh M, Wahi S, Kumar M, Ganguli A (2007). Prevalence of enterotoxigenic *Staphylococcus aureus* and Shigella spp. in some raw street vended Indian foods. Int J 54 55 Environ Health Res.

[17] Eromo T, Tassew H, Daka D, Kibru G (2016). Bacteriological quality of street foods and antimicrobial resistance of isolates in Hawassa, Ethiopia. Ethiopian J Health Sci.

[18] Chauhan N, Uniyal V, Rawat DS. Microbial profiling of street foods of different locations at Dehradun city, India. Int J Curr Microbiol App Sci. 2015;4(1):340-7.

[19] Estrada-Garcia T, Lopez-Saucedo C, Zamarripa-Ayala B, Thompson M, Gutierrez-Cogco L, Mancera-Martinez A (2004). Prevalence of Escherichia coli and Salmonella spp. in street-vended food of open markets (tianguis) and general hygienic and trading practices in Mexico City. Epidemiol Infect.

[20] Muleta D, Ashenafi M (2001). Salmonella, Shigella and growth potential of other food-borne pathogens in Ethiopian street vended foods. East African Med J.

[21] Ejeta G, Molla B, Alemayehu D, Muckle C (2004). Salmonella serotypes isolated from minced meat beef, mutton and pork in Addis Ababa, Ethiopia. Revue de médecine vétérinaire.

[22] King JC, Black RE, Doyle MP, Fritsche KL, Halbrook BH, Levander OA (2000). Foodborne illnesses and nutritional status: a statement from an American Society for Nutritional Sciences Working Group. J Nutr.

[23] Kumie A, Zeru K (2007). Sanitary conditions of food establishments in Mekelle town, Tigray, North Ethiopia. Ethiop J Health Dev.

[24] D. R. Practical food microbiology: 2003 (3rd edition).

[25] Collee JG. Mackie & Mccartney practical medical microbiology (14Th Edition). 1996.

[26] Brown JH. Bergey’s manual of determinative bacteriology. American Public Health Association; 1939.

[27] Cheesbrough M. District laboratory practice in tropical countries: Cambridge university press; 2006.

[28] UK Standards (2014). UK Standards for microbiology investigations identification of Enterobacteriaceae: bacteriology test procedures Standards UNIT. Public Health England.

[29] Oranusi S, Olorunfemi O (2011). Microbiological safety evaluation of street vended ready-to-eat fruits sold in Ota, Ogun state, Nigeria. Int J Res Biol Sci.

[30] Mackie TJ (1989). Mackie & McCartney practical medical microbiology: Churchill living stone.

[31] CLSI. Performance standards for antimicrobial susceptibility testing. 2017.

[32] (1997) WHO (World Health Organization). World Health Statistics Quarterly Geneva. Switzerland. 1997; 50(1):2;1(50):2.

[33] Rane S (2011). Street vended food in developing world: hazard analyses. Indian J Microbiol.

[34] Muleta D, Ashenafi M. Some Street Vended Foods From Addis Ababa: Microbiological and Socio-economic Descriptions. Ethiopian Journal of Health Sciences. 2000; 10(2).

[35] Tafesse F, Desse G, Bacha K, Alemayehu H (2014). Microbiological quality and safety of street vended raw meat in Jijiga town of Somali regional state, Southeast Ethiopia. Afr J Microbiol Res.

[36] Derbew G, Sahle S, Endris M (2013). Bacteriological assessment of some street vended foods in Gondar, Ethiopia. Int J Food Saf.

[37] Abdalla M, Suliman S, Bakhiet A. Food safety knowledge and practices of street food vendors in Atbara City (Naher Elneel State Sudan). African Journal of Biotechnology. 2009; 8(24).

[38] Chumber SK, Kaushik K, Savy S. Bacteriological analysis of street foods in Pune. Indian Journal of Public Health. 2007; 51(2). 18240473

[39] Bereda TW, Emerie YM, Reta MA, Asfaw HS (2016). Microbiological safety of street vended foods in Jigjiga City, eastern Ethiopia. Ethiopian J Health Sci.

[40] Edima H, Tem Nnam R, Awono Enama T, Biloa D, Ndjouenkeu R (2014). Case study of the street food sector in the metropolitan areas of a Cameroonian City, Yaounde. Int J Curr Microbiol App Sci.

[41] Asefa A, Bimerew M, Tadesse G, Gizaw Z, Adane T (2016). Bacteriological quality assessment of selected street foods and their public health importance in Gondar town, North West Ethiopia. Global Veterinaria.

[42] Kwiri R, Winini C, Tongonya J, Gwala W, Mpofu E, Mujuru F, et al. Microbiological safety of cooked vended foods in an urban informal market: a case study of Mbare Msika, Harare, Zimbabwe. 2014.

[43] Githaiga GM. Microbial quality, strain distribution and Enterotoxigenicity of selected food borne pathogens in relation to the hygienic practices in industrial area, Nairobi, Kenya: Faculty of Agriculture, College of Agriculture and Veterinary Sciences, University of Nairobi; 2012.

[44] Shojaei H, Shooshtaripoor J, Amiri M (2006). Efficacy of simple hand-washing in reduction of microbial hand contamination of Iranian food handlers. Food Res Int.

[45] Tabashsum Z, Khalil I, Nazimuddin M, Mollah A, Inatsu Y, Bari ML (2013). Prevalence of food borne pathogens and spoilage microorganisms and their drug resistant status in different street foods of Dhaka city. Agric Food Anal Bacteriol J.

[46] Sharma I, Mazumdar J (2014). Assessment of bacteriological quality of ready to eat food vended in streets of Silchar city, Assam, India. Indian J Med Microbiol.

[47] Akusu O, Kiin-Kabari D, Wemedo S (2016). Microbiological quality of selected street vended foods in Port Harcourt metropolis, Rivers state, Nigeria. Sky J Food Sci.

[48] Feglo P, Sakyi K (2012). Bacterial contamination of street vending food in Kumasi, Ghana. J Med Biomed Sci.

[49] Makelele L, Kazadi Z, Oleko R, Foma R, A Mpalang RK (2015). Microbiological quality of food sold by street vendors in Kisangani, Democratic Republic of Congo. Afr J Food Sci.

[50] Saranraj P. Microbial spoilage of bakery products and its control by preservatives. International Journal of Pharmaceutical & Biological Archive. 2012; 3(1).

[51] Odeyemi OA, Sani NA. Antibiotic resistance and burden of foodborne diseases in developing countries. Future Science; 2016. 10.4155/fsoa-2016-0023 PMC5242143 28116122

